# A Case of Pulmonary Sarcoma with Significant Extension into the Right Lung

**DOI:** 10.1155/2014/279374

**Published:** 2014-11-13

**Authors:** Yoshiaki Inoue, Yotaro Izumi, Kenjiro Sakaki, Keiko Abe, Teruaki Oka, Jun-Ichi Tamaru, Ato Sugiyama, Kohei Aoki, Hiroki Fukuda, Masatoshi Gika, Kazuhito Imanaka, Mitsuo Nakayama

**Affiliations:** ^1^Department of General Thoracic Surgery, Saitama Medical Center, Saitama Medical University, 1981 Kamoda, Kawagoe, Saitama 350-8550, Japan; ^2^Department of Cardiovascular Surgery, Saitama Medical Center, Saitama Medical University, Saitama 350-8550, Japan; ^3^Department of Pathology, Saitama Medical Center, Saitama Medical University, Saitama 350-8550, Japan

## Abstract

A female patient in her 30s was referred to us with a mass approximately 8 centimeters in diameter in right lung segment 6. Bronchoscopy was done, and a tumorous lesion obstructing right B6 was found. Biopsy of this lesion supported suspicions of sarcoma or spindle cell carcinoma. Contrast-enhanced CT showed that the mass extended to and obstructed the right main pulmonary artery. A skip lesion was also suspected in the periphery of pulmonary artery trunk. The tumor was removed by right pneumonectomy accompanied by resection of the main and left pulmonary arteries under cardiopulmonary bypass. The pulmonary artery trunk and the left pulmonary artery were reconstructed with a vascular graft. Collectively, intimal sarcoma originating from the right main pulmonary artery with extension into the right lung was diagnosed. Significant extension of pulmonary artery sarcoma into the lung, as was observed in the present case, is considered to be rare, and to our knowledge this is the first report in which the primary lesion was biopsied by bronchoscopy.

## 1. Introduction

Sarcoma of the pulmonary artery (PAS) is a rare disease with about 200 cases reported so far in the literature [1, 2]. The prognosis is generally poor with a reported mean survival of about 20 months [1, 2]. In most cases, the lesion is confined to the vicinity of the main pulmonary artery. Here we report a case of PAS with significant extension into the right lung.

## 2. Case Presentation

**Figure 1:**
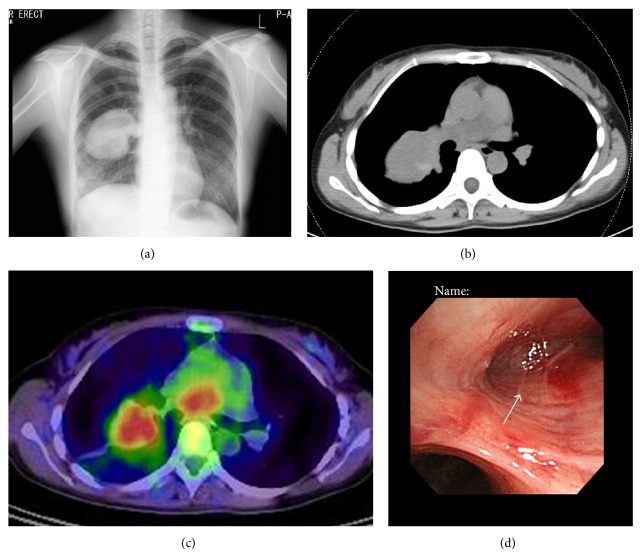
(a) Chest radiography showing a mass in the right middle lung field. (b) Noncontrast CT showing a mass approximately 8 centimeters in diameter with relatively smooth margins in the right lung segment 6. (c) Fluorodeoxyglucose-positron emission tomography showing accumulation in the right lung mass and in the lesion in the mediastinum. (d) On bronchoscopy, a whitish tumorous lesion was seen obstructing right B6 (arrow).

A female patient in her 30s was referred to our institution with a complaint of persistent cough. Chest radiography revealed a mass in the right middle lung field (Figure 1(a)). Noncontrast CT showed a mass approximately 8 centimeters in diameter with relatively smooth margins in the right lung segment 6 (Figure 1(b)). A malignant lung tumor was suspected and fluorodeoxyglucose-positron emission tomography (FDG-PET/CT) was done. FDG accumulation was seen in the lung mass (standardized uptake value max. 3.6) as well as in the mediastinum suggesting a malignant lung tumor with mediastinal lymph node metastasis (Figure 1(c)). Bronchoscopy revealed a tumorous lesion obstructing right B6 (Figure 1(d)). Biopsy of this lesion showed proliferation of spindle cells raising suspicion of sarcoma or spindle cell carcinoma.

**Figure 2:**
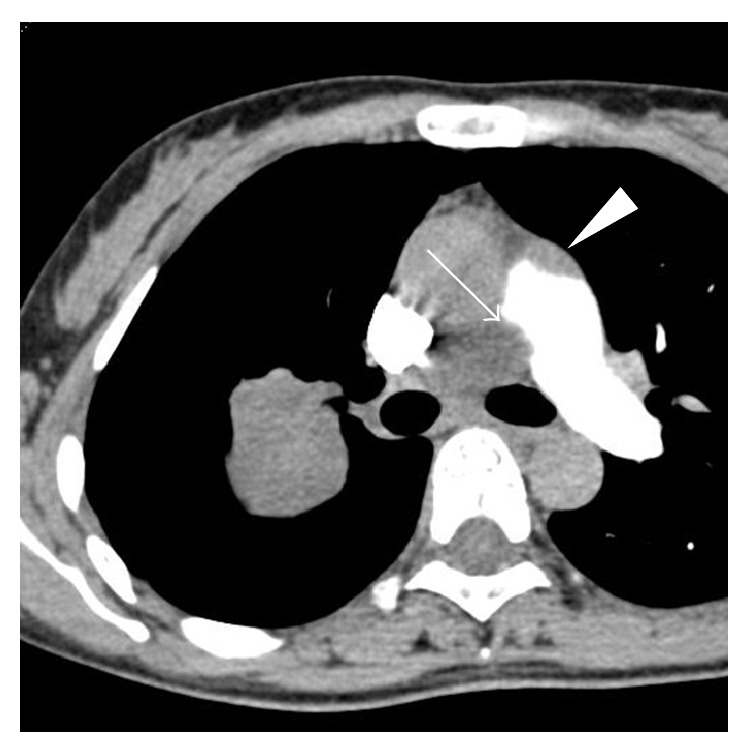
Contrast-enhanced CT showing obstruction of the right pulmonary artery (arrow). A skip lesion was also suspected in the periphery of the pulmonary artery trunk (arrowhead).

Next, contrast-enhanced CT was done, and it showed that the mediastinal lesion pointed out on FDG-PET/CT was actually a continuous extension of the lung mass into the right main pulmonary artery. A skip lesion was also suspected in the periphery of the pulmonary artery trunk (Figure 2).

Lung ventilation-perfusion scan showed that the right lung was not perfused although sufficiently ventilated. On echocardiography, heart wall motion was intact and no signs of right heart failure were seen.

Collectively, a sarcomatous lung tumor with extension into the right pulmonary artery trunk or PAS with extension into the right lung was suspected. The patient underwent surgical resection of the tumor under cardiopulmonary bypass through a median sternotomy. The existence and localization of the skip lesion was first confirmed by transthoracic and transesophageal echocardiography. Venous cannulas were placed in the right atrium and the femoral vein, and an arterial cannula was placed onto the femoral artery, and surgery was carried out with the heart beating. The left pulmonary artery was carefully dissected so as not to perturb the skip lesion and was clamped just proximal to the origin of left A3. Although the tumor filled the inside of the right pulmonary artery, its wall appeared to be intact from outside. Then the main pulmonary artery was incised just above the pulmonary valve and opened distally to expose the tumor. To remove all lesions, proximal portion of the left pulmonary artery together with the pulmonary artery trunk was resected. The left pulmonary artery was reconstructed with a 20 mm Gore-Tex vascular graft and the patient was separated from cardiopulmonary bypass. Following that, right pneumonectomy and total resection of the tumor were completed. Time on bypass was 2 hours and 11 minutes. Duration of operation was 9 hours and 9 minutes.

**Figure 3:**
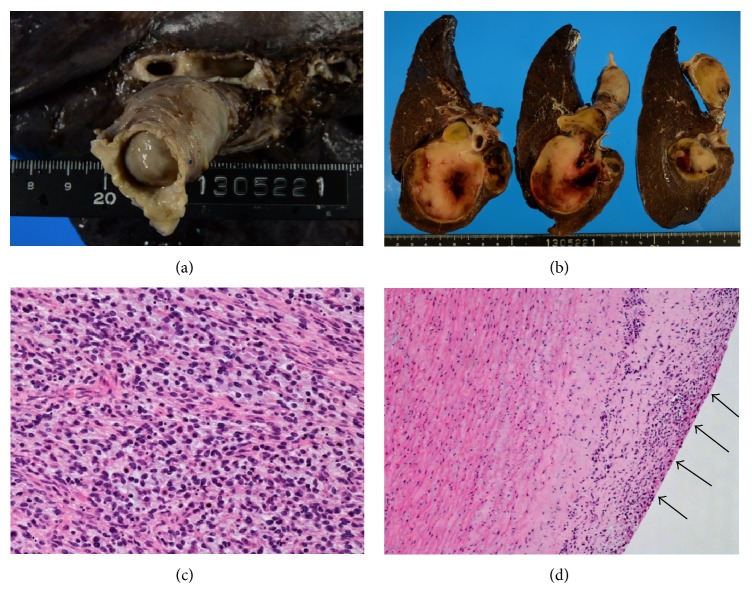
(a) Gross finding showed the obstruction of the right main pulmonary artery by tumor tissue. (b) Transverse sections revealed a massive invasion of the tumor into the adjacent pulmonary parenchyma. (c) Histologically, tumor was consisting of spindle or polygonal-shaped atypical cell with high mitotic activity, compatible with intimal sarcoma. (d) At the margin of the left main pulmonary artery, pleomorphic tumor cells proliferated in the vascular intima (arrows).

Grossly, the tumor seemed to arise in the large vessel of the pulmonary circulation and invade into the adjacent pulmonary parenchyma, especially in the S6 region (Figures 3(a) and 3(b)). The tumor was diagnosed as intimal sarcoma and its histological findings, proliferation of atypical spindle or polygonal cells with high mitotic activity, were also compatible with the diagnosis (Figure 3(c)). The lesions were completely removed macroscopically. Further resection, particularly the distal portion of the left main pulmonary artery, was not considered to be feasible. However, the surgical margin of the left pulmonary artery was microscopically positive for tumor cells (Figure 3(d)). The patient was followed up without further treatment.

Twelve months after surgery, recurrence in the right ventricular outflow tract was detected. Irradiation and proton therapy were done. The recurrence is without progression 2 months after detection.

## 3. Discussion

Clinical diagnosis of PAS is considered to be difficult. Symptoms such as cough, dyspnea, chest or back pain, fever, or weight loss have been reported, but they are usually nonspecific. Previous reports have shown that the lesions are mostly confined to the vicinity of pulmonary artery trunk and are often initially diagnosed as pulmonary thromboembolisms. FDG-PET may be useful in differentiating pulmonary thromboembolism and PAS, but some reports indicate that PASs may not always show high FDG uptake [3, 4]. Case reports of EBUS-TBNA exist showing its effectiveness in the differential diagnosis [5, 6].

In the present case, a malignant lung tumor with mediastinal lymph node metastasis was initially suspected from noncontrast CT and FDG-PET/CT. To our knowledge, significant extension of PAS into the lung, as was observed in the present case, has not been previously reported. This is also the first report of PAS in which the primary lesion could be biopsied under bronchoscopy. The reason for this tumor extension is not clear. It is possible that in this particular case the primary lesion originated from the right main pulmonary artery rather than the pulmonary artery trunk. Since PASs are generally known to extend in the direction of blood flow [7], the lesion in the present case extended predominantly towards the periphery of the right pulmonary artery rather than towards the pulmonary artery trunk, and, because of this unilateral extension, the patient remained symptomless until a large tumor was formed in the right lung.

PAS may show a variety of histological differentiations such as leiomyosarcomas, angiosarcomas, malignant fibrous histiocytomas, and others but are mostly without differentiation. These are collectively termed intimal sarcomas, as is this case [1, 8]. Early diagnosis and surgical resection currently offer the best chance of prolonged survival for PAS. The efficacy of chemotherapy or radiotherapy for PAS still remains controversial [8, 9]. Therefore, adjuvant therapy was not done in this case although the surgical margin was positive. After the detection of recurrence, proton therapy was selected because the recurrence was localized, and we considered that the general condition of the patient would be best sustained by this treatment in comparison with systemic chemotherapy or reresection.
